# Artificial Intelligence, Machine Learning and Smart Technologies for Nondestructive Evaluation

**DOI:** 10.3390/s22114055

**Published:** 2022-05-27

**Authors:** Hossein Taheri, Maria Gonzalez Bocanegra, Mohammad Taheri

**Affiliations:** 1 Laboratory for Advanced Non-Destructive Testing, In-Situ Monitoring and Evaluation (LANDTIE), Department of Manufacturing Engineering, Georgia Southern University, Statesboro, GA 30458, USA; mg07926@georgiasouthern.edu; 2 Department of Mathematics and Statistics, South Dakota State University, Brookings, SD 57007, USA; mohammad.taheri@jacks.sdstate.edu

**Keywords:** nondestructive evaluation (NDE), Artificial Intelligence (AI), machine learning (ML), NDE 4.0, digital twins

## Abstract

Nondestructive evaluation (NDE) techniques are used in many industries to evaluate the properties of components and inspect for flaws and anomalies in structures without altering the part’s integrity or causing damage to the component being tested. This includes monitoring materials’ condition (Material State Awareness (MSA)) and health of structures (Structural Health Monitoring (SHM)). NDE techniques are highly valuable tools to help prevent potential losses and hazards arising from the failure of a component while saving time and cost by not compromising its future usage. On the other hand, Artificial Intelligence (AI) and Machine Learning (ML) techniques are useful tools which can help automating data collection and analyses, providing new insights, and potentially improving detection performance in a quick and low effort manner with great cost savings. This paper presents a survey on state of the art AI-ML techniques for NDE and the application of related smart technologies including Machine Vision (MV) and Digital Twins in NDE.

## 1. Introduction

### 1.1. Nondestructive Evaluation (NDE)

Nondestructive Evaluation (NDE) is an accepted and well-established method of inspection, material state awareness (MSA), structural health monitoring (SHM) and in situ process monitoring for almost every part and product during manufacturing processes and service life of the components [1,2,3]. State-of-the-art and future of NDE requires a significant increase in accuracy, speed of both inspection and data processing, and reliability but at lower cost such that NDE can catch up with the advancements in manufacturing, advanced materials (such as composites and powder metallurgy), infrastructure and other relevant technologies [4,5,6]. In addition, the applications of robotics and automation in NDE have been increased significantly to reduce inspection time, reduce human error, improve probability of detection (POD) and facilitate the interpretation of NDE results [4,7,8].

### 1.2. Artificial Intelligence

NDE techniques require high level of intelligence and discernment in performing the experiments and interpreting the results. Artificial Intelligence (AI) which is the intelligence demonstrated by machines to do tasks is a well-suited tool for NDE applications [9,10]. Major elements which drive the widespread application of AI algorithms include: broader development and availability of algorithms which some of them are open source and easy to use, availability of large sets of data for training, development and advancement of computational devices and their capabilities, and strong interest in new technologies such as smart manufacturing, autonomous devices and automated data processing [11].

## 2. Artificial Intelligence in NDE

**Figure 1:**
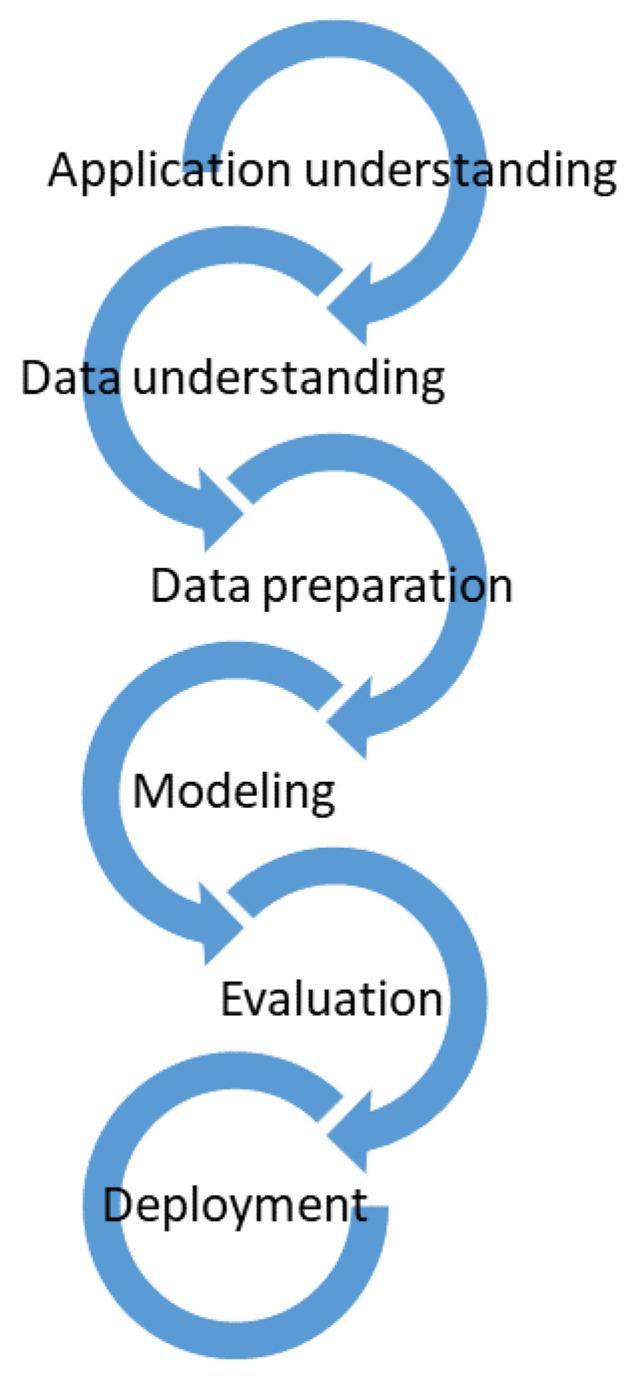
Process model for application of AI in NDE.

The requirements due to advances in NDE technologies and NDE automation implies the crucial need for consistent and accurate evaluation of test results in terms of signals, data, images and patterns. To address these emerging needs, an intelligence knowledge-based system is desired such that it can take the NDE testing results or data, and produce an intelligent output in the form of classified and systematic interpretation of the results. AI methods are promising and capable ways for the goals of automated and efficient evaluation of NDE data and test results. AI methods and algorithms have been recently used with success in various NDE, SHM and predictive-preventive maintenance applications [11]. Shrifan et al. discussed prospect of using AI for microwave NDE [12]. According to Shrifan, AI techniques combined with signal processing techniques are highly possible to enhance the efficiency and resolution of microwave NDE and have tremendous potential and viability for evaluating structures quality. Another main reason which makes AI a perfect tool for NDE and specifically automated NDE is that considering the large amount of complex signals and data from the NDE inspection, nobody needs to really study the details of the physics or manufacturing process to understand what the correct process parameters and the quality of the product are [13]. Instead, AI algorithms aim to identify, by looking at training and/or test data, if the process or the part has passed the quality criteria. This is particularly useful for the operation and operators. However, development of AI algorithms in NDE requires the involvement of expert knowledge in each and every step in a systematic approach such as the flowchart shown in Figure 1.

### 2.1. Machine Learning

Machine learning (ML) is a subsection of the broad field of AI. This field aims to mimic the learning and recognition abilities of the human brain as well as the ability of self-optimization. The first person to formulate and use the term “Machine Learning” was the computer gaming and artificial intelligence pioneer, Arthur Samuel in 1959. He established that machine learning is an algorithm that is able to capture associations and learn patterns from data without enforcing specific and explicit code instructions [14]. In other words, without prior information on how to recognize latent relationships in the data. This methodology is highly used when met with high-dimensional datasets given its use of statistics and large training datasets. NDE has a special interest in ML algorithms because of their ability to automate tasks, in this case, the structural health monitoring and condition assessment of materials. Statistical ML techniques can be used for prediction of defect’s characteristics based on known data set of defects due to their capabilities in estimating unknown values based on training data set [15].

The desire to use ML algorithms in NDE stems from the need to have a precise prediction method to detect defects in materials and structures. With this in mind, the defect analysis and detection systems have to keep up with the increased industrial production of materials. Additionally, these systems should be as precise as possible and with minimum margin of error, including human error. The more data used to train the algorithms, the better it gets at predicting and classifying NDE data.

There exist four different subsections of Machine Learning which are: Unsupervised Learning, Supervised Learning, Semi-Supervised Learning, and Reinforcement Learning. These sections are separated based on how the data is fed into an ML algorithm and how it learns from that data. Supervised and Unsupervised learning methods are the most used and applicable methods for NDE applications.

### 2.2. Unsupervised Learning

Unsupervised learning refers to the process of feeding unlabeled and unclassified data into an ML algorithm to extract patterns. The algorithm is expected to learn these patterns and underlying relationships to properly detect similarities and disparities of groups in the data without human intervention. Unsupervised learning algorithms are typically more difficult to train than Supervised Learning ones, clearly from their nature of minimal human interaction. For example, unsupervised learning algorithms cannot be applied to classification or regression problems. This stems from the need of an output target, which is unknown to the algorithm given it has no idea what it is looking for. The algorithm itself is merely trying to find common characteristics the data and grouping it. Using unsupervised learning algorithms is advantageous when trying to gain insight on the available data. However, the algorithms are not perfect and are prone to produce erroneous associations given that there is not target output. Unsupervised learning also partitions itself into two subsections: Clustering and Association.

### 2.3. Cluster Analysis

Clustering is the task of dividing the population or group of data into different groups or clusters. This method stores data points that are the most similar, together. The data point’s similarity is based on underlying features and patterns that are meaningful to the clusters. There currently exist various methods of cluster analysis, such as Density-Based Clustering, Hierarchical Clustering, K-Means Clustering, and Spectral Clustering.

#### 2.3.1. K-Means Algorithm

**Figure 2:**
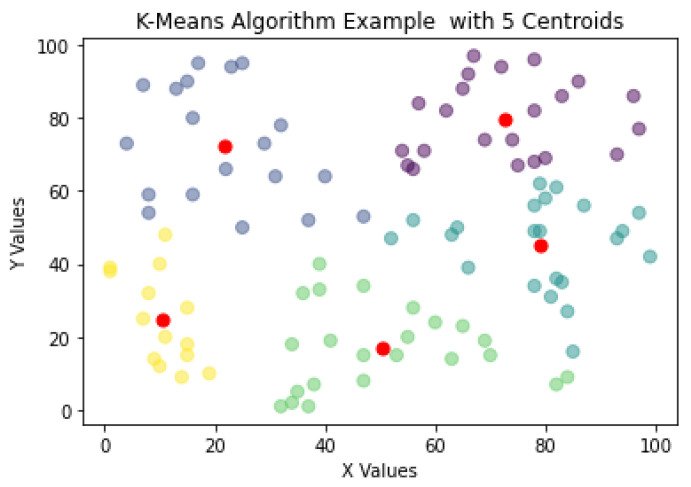
K-means algorithm plot example with random data.

One of the most used algorithms for NDE is the K-means algorithm. When presented with an unlabeled dataset, the K-means algorithms partitions the data in k clusters defined by a centroid. A cluster is defined as a collection of data points placed together in space due to similar characteristics shared by the data points. Furthermore, the centroid is the location of the cluster’s center. It is defined that with the K-means clustering, the plane or data space will always form a Voronoi diagram. Figure 2 shows how the K-means algorithm would create clusters around a centroid, in this case k = 5 with the centroids marked as red data points. In [16], the authors applied the K-means cluster to classify acoustic emission signals obtained from analyzing the stress-corrosion-cracking (SCC) on 304 nuclear grade stainless steel. The authors of [16], determined that each data point in the cluster was composed of five signal characteristics: ring count, duration, rise time, amplitude, and energy. Based on observations done by the authors, Du et al. [16] experimented with the use of three clusters (k = 3) which represented types damages encountered in the steel (crack propagation, pitting, and bubble break-up). Through the resulting data clusters found by the K-means algorithm, Du et al. [16] concluded that the clusters were mainly defined by the amplitude and the frequency band energy. Other studies have also explored the use of K-means clustering in other NDE applications. In [17], the algorithm is used for defect detection in thermal images of industrial materials, while in [18] the K-means is leveraged for defect detection when using pulse eddy currents. The K-means algorithm has proven to be a powerful tool and is still predominantly used in the area of NDE.

#### 2.3.2. Density-Based Clustering

**Figure 3:**
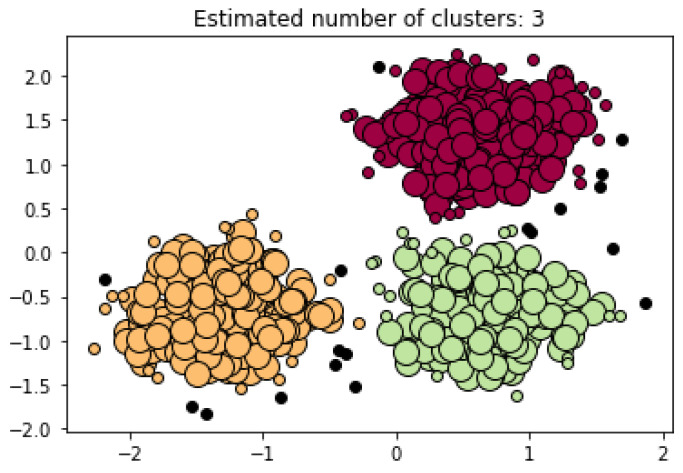
DBSCAN Plot Example with 3 Dense Clusters (After [22]).

The Density-Based Clustering (DBC) algorithm is contingent on the idea that datasets contain dense data point regions separated by low regions of data points. In contrast to the K-means algorithm, the DBC algorithms do not need a predetermined k value. Discarding the k value allows for the DBC algorithms to find the number of clusters present in a dataset by analyzing the density distribution of data points in space. An advantage of these algorithms in comparison to the K-means is that it can discern noise and outliers in the data. The noise and outliers are presented as low regions of data point density. In other words, the K-means algorithm would pull these outliers into a cluster due to its k constraint while the DBC would inherently leave them alone. Regarding the use of the DBC algorithms in NDT, many papers predominantly use a branch of the DBC which is the Density-Based Spatial Clustering Applications with Noise (DBSCAN). The DBSCAN leverages a data point’s density reachability and density connectivity [19]. The density reachability specifies the maximum distance of two data points to be considered neighbors and part of the same cluster. On the other hand, the density connectivity specifies the minimum number data points needed to define a cluster in a region of space. The fields of Civil and Manufacturing Engineering predominantly use the DBSCAN algorithm to monitor the structural health of materials. The study in [20] explores Ultrasonic Testing (UT) to inspect the pressures of tubes in the of the Ontario Hydro. Canada’s system, Channel Inspection and Gauging Apparatus for Reactors (CIGAR) uses UT to obtain volumetric images of the pipelines and assess defective regions in them. The statistical properties of the ultrasonic signals are mapped as data points in space, from which the DBSCAN can conduct its analysis [21]. An example of the DBSCAN algorithm in use can be observed in Figure 3. In this case, the algorithm constructed an analysis based on three clusters created by three dense regions encountered in the dataset. Furthermore, in this example the DBSCAN found 18 points of noise in the dataset.

#### 2.3.3. Spectral Clustering

**Figure 4:**
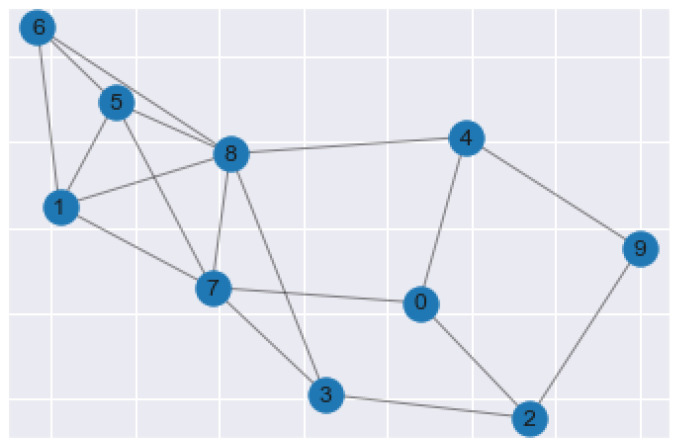
Spectral Clustering Plot Example.

Spectral Clustering is the machine learning method in which the data points in the data set are treated as nodes of a graph. This algorithm is rooted in graph theory and is treated as a graph partitioning problem. The end goal of spectral clustering is to cluster data that is connected but not necessarily in round cluster shapes, as seen in Figure 4. The nodes, or data points, in a data set are projected into a low-dimensional space where connectivity clusters can be achieved. The first step of conducting this algorithm is to compute a similarity graph. The similarity graph is analogous to cluster formation.

In the paper [23], the authors apply terahertz time-domain spectroscopy imaging to conduct nondestructive evaluation tests. This method is applied on three industrial ceramic matrix composites and one defective silicon slice. The spectral image produced by the analysis is used to detect defects such as superficial damage or internal bubbles. The spectral data is then applied to the spectral clustering recognition algorithm.

#### 2.3.4. Hierarchical Clustering

Hierarchical clustering is an analytical algorithm used to compute clusters of data points. This type of clustering algorithm is subdivided into two different techniques, agglomerative and divisive. The agglomerative technique imposes that each data point starts as its cluster. Furthermore, the algorithm computes a proximity matrix from each point in space. In every iteration, the algorithm merges the two closest pairs of clusters and a new proximity matrix is computed.

**Figure 5:**
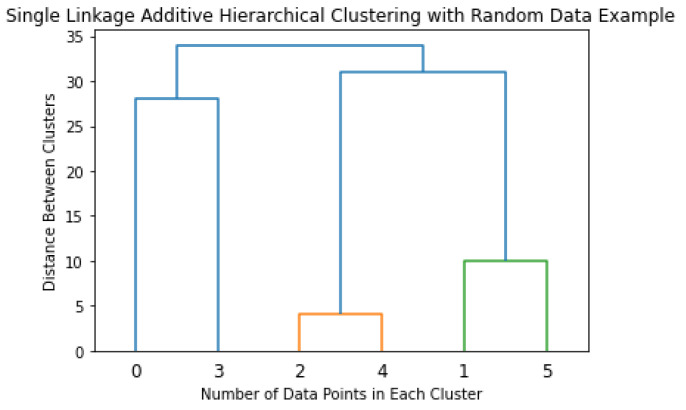
Single Linkage Additive Hierarchical Clustering with Random Data.

There exist four forms of cluster linkage to conduct additive hierarchical clustering. Complete linkage computes the similarity of the furthest pairs in the dataset; however, it is prone to errors if there exist outliers. Single linkage works similarly, but it conducts this comparison between the closest data points. In the same fashion as the previous linkage processes, centroid linkage compares the centroids of each cluster and merges them given found similarities. The last linkage method, group average, finds similarities between the overall clusters and merges them. The linkage process is repeated until a predetermined number of clusters is achieved. The optimal way of describing the additive hierarchical clustering is by creating a dendrogram, as shown in Figure 5.

In contrast to additive clustering, divisive clustering works by starting with all data points in space belonging to one cluster. In this case, the algorithm will split away from the furthest points in the cluster until the predetermined number of clusters is achieved. Divisive clustering is not commonly used for NDE.

The application of additive hierarchical clustering in NDE can be observed in [24] through the implementation of ultrasonic echo testing. Several sensors are implemented on the material to obtain key feedback from ultrasonic echo testing. The data is then fed to independent component analysis mixture models (ICAMM) to identify all possible defects that can be encountered in the material. In the case of [24], the authors implement a single linkage after the ICAMM model completes its analysis. The hierarchical cluster algorithm is then tasked to identify and link clusters of similar defects based on the data points provided. The experimentation done in [24] included 5 different categories of defects. From these categories, four of them belonged to single-defect materials, and one was composed of multiple defects. Their results concluded that the algorithm was successful at creating a hierarchy with significant separation of the one-defect materials and multiple defect materials.

### 2.4. Association Analysis

Association analysis is an unsupervised learning method that seeks to find underlying patterns and relationships to describe the data at hand. Association analysis is mainly used to find frequent patterns between different variables in a data set. These patterns can be found based on frequency of complimentary occurrences among variables. The descriptive nature of the association analysis algorithms allows for a better understanding of the data and ties into the area of feature engineering and extraction. Association analysis is frequently implemented with common statistical methods. Common association analysis deals with the occurrence of one variable with another. The process seeks to find frequent patterns that can help explain the data.

Due to its characteristics, association analysis is a useful technique for discovering interesting relationships hidden in large data sets. Such situations is common in recent methods of continuous monitoring NDE and SHM. Through recent advancements in sensors and sensing technologies such as distributed fiber optic sensors and multi-point laser vibrometers for SHM, or even acoustic emission sensors and data acquisition units for manufacturing process monitoring [25], it is possible to acquire large data sets which often contain uncovered relationships in features of the data, useful for structural integrity assessment. Engineers, designers and technicians can use this type of relations and conclusions to establish enhanced predictive and preventive maintenance plans.

### 2.5. Supervised Learning

Supervised learning is a branch of ML in which the user feeds labeled and classified data to the machine learning algorithms. This data-input methodology helps the algorithm to detect the relationships and patterns faster. Supervised learning is often divided into different categories based on the desired output. The first category in supervised learning is the algorithms with a discrete classification end goal. These algorithms are trained to differentiate among different classification categories from which they were previously trained. The input is analyzed by the supervised learning algorithm and placed in a category that the algorithm finds more suitable given the information extracted from the input (show a feature map and classification examples, very general). The second category of supervised learning is made of algorithms that produce a continuous output (e.g., Regression Analysis) [15].

#### 2.5.1. Support Vector Machine

**Figure 6:**
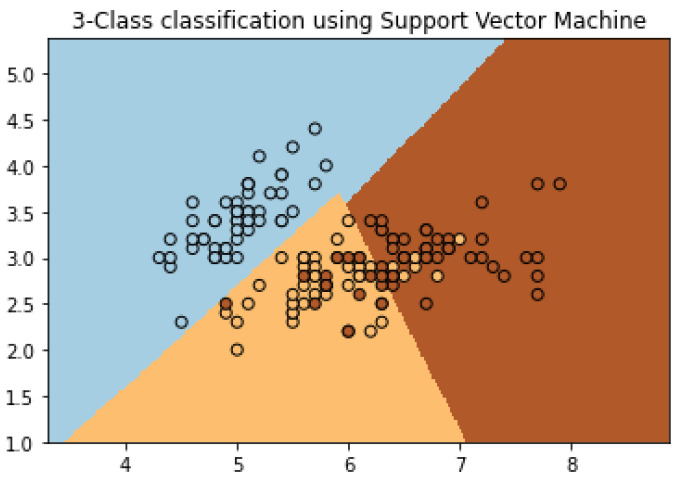
Support Vector Machine Example (After [22]).

One of the techniques in non-destructive testing that classifies and at the same time obtains a regression from the input data is the Support Vector Machine (SVM) algorithm. This algorithm creates support vector representations from fed labeled examples to properly classify said labels. The support vectors for each class are the most difficult points to classify since they are the closest to the boundary that separates those classes. The separating hyperplanes serve as the plane that separates data into different classes and is highly effective in multidimensional data classification (e.g., two or more classes). One can observe the hyperplane as a line that separates the data in a two-dimensional plane into three different classes, as seen in Figure 6. This line can then be expanded into higher dimensionalities, e.g., a hyperplane, when more classes are introduced in the data. Moreover, there exist many hyperplanes that can separate the data using the support vectors, nonetheless, there is only one hyperplane that has the optimal distance with each support vector. Employing the most optimal hyperplane allows for better data representation and classification outputs. However, sometimes the most optimal hyperplane is not the best representation of the data. In this case, the SVM introduces the kernel function. The kernel function adds dimensionality to the data by employing mathematical functions on the original data and creating a data set of higher dimensionalities. Then, the SVM algorithm is used in the higher dimensionalities data set to find the most optimal hyperplane [26].

Support vector machine algorithms have been extensively used in numerous NDE research [27]. The study [28], talks about the use of SVM for X-ray casting inspection. The X-ray images used are to analyze weld defects by inputting them into a sorting algorithm. The sorting algorithm casts a wide net of possible defects found in the images and sends these images to the SVM. The SVM will sort the X-ray images into likely and unlikely images of defects. The input analyzed features for the SVM are (1) the area of pixels of potential weld defects and (2) the average parallel greyscale pixel difference to weld edge joints [28]. This method allows for the automation of fault detection. Other studies, like [29], have developed NDE methods using the Long-Range Ultrasonic Testing technique. In [29], the authors develop a method of defect detection in oil and gas pipelines to prevent future failures or unscheduled shutdowns. The classification is produced through the combination of the SVM and the calculation of the average Euclidean distance between the testing data points and the previously defined support vector set for each category. The closest set defines the classification output for the data point. In this case, the SVM allows for continuous monitoring of oil and gas pipelines without much human intervention from operators.

#### 2.5.2. K-Nearest Neighbor

The K-Nearest Neighbor (KNN) algorithm relies on the idea that similar data points in a data set are going to be close to each other in space, in other words; neighbors. KNN falls under supervised learning algorithms, however it can perform both regression and classification problems. The KNN algorithm assumes that point proximity represents class similarity. The number of points necessary to determine the output classification label of a data point is established by the hyperparameter k, which is not to be confused with the k from clustering analysis techniques. The closest k points to the query input point have their own label and this will help determine the output label of the query point.

The measuring technique used to measure the distance between two points defines if KNN is being used for categorical or quantitative data. To measure the distance between two categorical values, the algorithm implements the Hamming distance measurement. On the other hand, KNN implements three possible distance functions for quantitative measurement. The Euclidean function is one of the most common ones, followed but the Manhattan function and then the Minkowski function.

In [30], the authors conducted structural health testing for coated/uncoated metallic or dielectric materials using microwave NDE. The focus was to search for cracks in the material using microwaves from 8.2 GHz to 12.4 GHz. The paper used feature extraction to first clean the data obtained from the microwave sensor. This method helped cleanse the data set of redundant data and improve learning accuracy with the most important features. The waves showcased a horizontal wave shift to the left when a crack was encountered in the material. With this information in mind, feature selection algorithms were implemented to choose the top five features that are leveraged to accurately detect cracks in the material. The features are then fed to the KNN algorithm for as data points to automate the crack detection process. The KNN algorithm yielded a 99.64 accuracy in crack detection. In [31], several ML models including KNN were applied to classify and predict the ultrasonic signals as degree of thermal aging of cast austenitic stainless steel (CASS). It was shown that for signals having large peak-to-peak amplitude, KNN shows higher classification performance than other classification models. Obvously, application of any particular classification models including KNN must be based on the case specifications and experience of the persons with both NDE and ML techniques.

#### 2.5.3. Neural Networks

**Figure 7:**
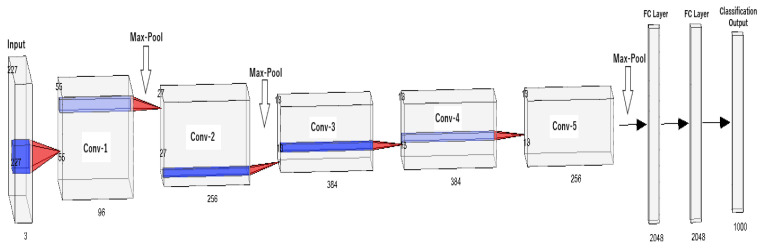
AlexNet Architecture.

Neural networks are machine learning algorithms inspired by the workings of the biological neurons of the human brain. This approach is also referred as “deep learning”. These algorithms are built upon stacking node layers made up of an input layer, output layer, and hidden layers. All individual nodes in a node layer have a specific value that activates them. Once a node is activated, it will send information to next node layer. The connections between the nodes are represented by a number, also known as the weight. The weight also holds influence on the nodes, meaning the higher the weight the more leverage it has on the network. In many cases, the neural network has a fully connected design which allows feed forward and feedback information. With this structure, the neural network can deduct information and improve itself based on previous decisions. One of the pillar neural networks in Artificial Intelligence is the LeNet 5 network created by Yann LeCun, et al. [32]. This network was developed to recognize handwritten digits and has been a base for many new and emerging networks in the field. Stemming from the same branch as LeNet 5, the famous Convolutional Neural Networks (CNN) have emerged. The widely popular AlexNet [33], a CNN variation, has been implemented across several fields to understand nonlinear data for classification. Figure 7 showcases the architectural design of AlexNet.

The application of neural networks in NDE is an emerging technique to assess the structural design and performance of materials. Specific work in NDE with the implementation of neural networks can be explored in [34]. In this paper, the authors develop a new experimental method to detect crack conditions of concrete surrounding reinforced steel. The new method implements the use of Ultrasonic Pulse Velocity test (UPV). Furthermore, with parameter control, UPV does not meaningfully impact crack propagation. The data obtain from the UPV is then fed to an Artificial Neural Network (ANN) to train and test. The ANN in [34] is trained on 100,000 cycles to achieve convergence and an optimal network without overfitting the data. In the case of [34], the ANN is treated as a black-box model to resolve pattern in the non-linear relationships encountered in the data. Each data point consists of four input variables that associate with the crack width. The ANN model intakes the strength of the concrete, the results of the UPV, concrete over reinforcement ratio, and path length where the UPV is set to travel [34]. Results obtained from the ANN showcased a strong relationship that variates based on the concrete strength of the concrete bond and the UPV. The increase of UPV shows a stronger bond condition of the material. The use of the ANN also proved that UPV is delayed by the presence of microfractures in the structure and thus help forecast the crack width in steel-concrete bonds. As research in NDE using neural networks is expanded, such as in [34], it becomes easier to pinpoint cracks and deterioration of materials.

The implementation of neural networks in NDE is also explored in the NDE technique of thermograms. In the case of [35], the authors explore a neural network approach to quantify depth defects in carbon fiber-based composites. Infrared thermograms help capture material irregularities by leveraging the different thermal responses. These thermal response profiles also help calculating the depth of the defects encountered. Given the multidimensional aspect of the thermal data obtained, the authors implement a neural network to search for the underlying relationship of the irregularities and the thermal response. The study in [35] implements standardized thermal contrast curves that represent defects of different sizes and locations which are used to feed into the input layer of the neural network.

### 2.6. Feature Extraction

A common technique in data analysis is feature extraction, which allows for dimensionality reduction of a data set. Moreover, a feature is an individual measurable property in a data set, such as length, width, height, etc. Data sets nowadays contain more and more features that intertwine with each other. However, when these data sets are fed into a machine-learning algorithm to train it, the algorithm will show overfitting when tested with unseen data before. With this in consideration, feature extraction aims to reduce the number of features or dimensions of a dataset by creating new features from existing ones. The new features are combined to create representations of the old features, which can now be discarded. This process also allows for the removal of data redundancy. Many machine learning algorithms implement feature extraction to reduce the dimensionality of their datasets. These algorithms include Random Forests, Artificial Neural Networks, and Autoencoders.

### 2.7. Machine Vision

The field of computer vision has experienced a boom in the past decade, with examples such as Facial & Object Recognition and Text Recognition. Moreover, this field is now joining Nondestructive Evaluation to automate the industrial and manufacturing processes. The use of computer vision algorithms helps the industry maintain and increase manufacturing quality. The analysis of information coming from images provides an easier and faster way to detect material flaws and avoid human error. Inspection systems using computer vision are designed to detect flaws with high precision and at faster rates than what human inspectors can. Furthermore, the algorithms profit from having scalability, meaning they can be used for large or small input datasets. Computer vision models also have the advantage of finding and leveraging feature importance to accurately detect flaws. Testing techniques that use these types of computer vision algorithms include X-rays, Thermal Images, Light Cameras, and Fluorescent Penetration Inspection to name a few. Image processing has allowed the manufacturing industry to further enhance their quality processes and automate the industry. In [36], 3D machine vision methods for pose (spatial position and orientation) estimation is investigated where an RGB-D camera observes the asset under inspection along with the probe; 3D machine vision processes the camera data to actively track the probe in relation to the asset, which further allows one to augment each NDE dataset with its inspection location.

In addition to NDE applications [37,38], Machine vision has also paved its way in SHM applications [39,40]. 3D visualization models can be generated to simulate the defect development in structure and infrastructures using machine vision techniques. In [41], spalling distress defects in subway networks were detected and quantified using image data processing and machine vision. Spalling is a significant surface defect that can compromise the integrity and durability of concrete structures. The core idea behind this technique was to create a complementary scheme of image preprocessing which is effective in isolating the object of interest from the background of the image.

## 3. Internet of Things (IoT)-Related Applications and NDE 4.0

**Figure 8:**
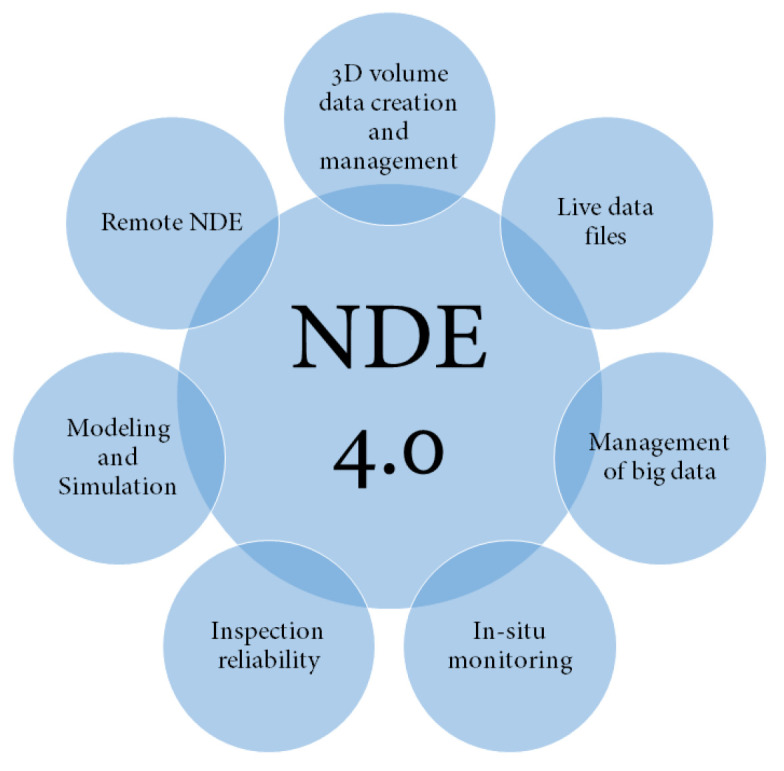
Aspects of new trends in NDE 4.0 (After [43]).

Digital transformation has been widely discussed in many different areas of industries [42] and it is finding its way into NDE applications. The term of NDE 4.0 was first introduced by Professor Norbert Meyendorf who introduced the term NDE 4.0 where the Internet of Things (IoT) and cyber physical systems are making a revolution in NDE industry [43]. In the concept of IoT, anything with an IP address can be connected through the internet as stated by Internet Protocol version 6 (IPv6). According to this concept by Meyendorff [44] and due to advancements in the era of Industry 4.0, the aspects of NDE reliability and human–machine interactions must be reconsidered such that NDE is also revolutionized to NDE 4.0 for Industry 4.0 [45]. Identified trends in NDE 4.0 are summarized in Figure 8.

**Figure 9:**
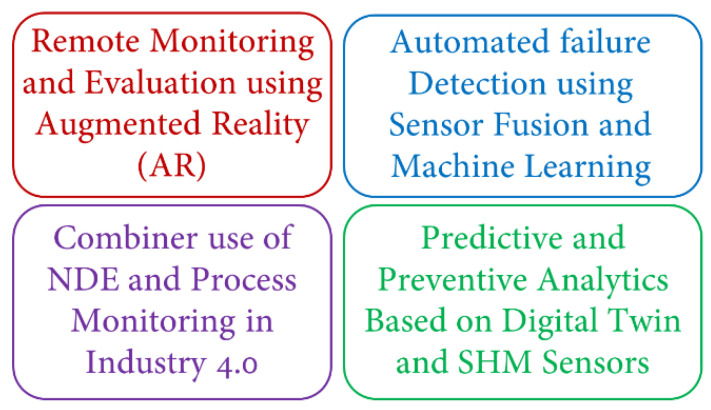
Core areas for digital transformation in the field of NDE as defined for Fraunhofer IKTS (After [11]).

Four core areas for digital transformation in the field of NDE have been identified by the Material Diagnostics branch of Fraunhofer IKTS (until 2014 Fraunhofer IZFP-D) as shown in Figure 9 [11].

**Figure 10:**
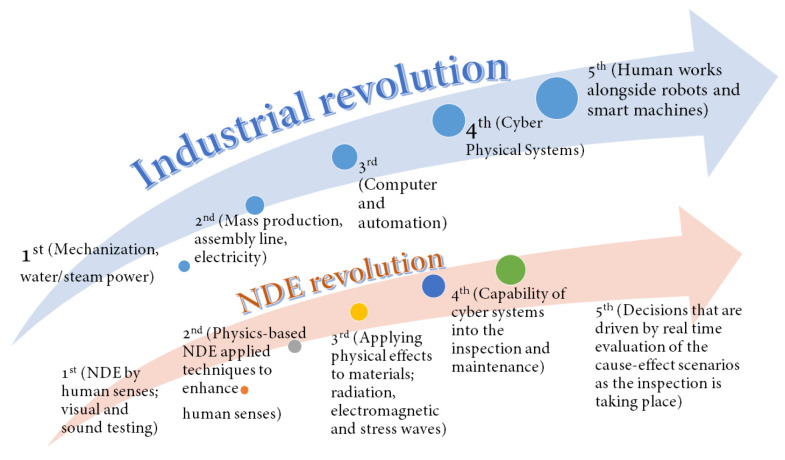
Similarities between industrial and NDE revolution steps.

As can be seen from the trends in NDE 4.0 and the identified core areas of digital transformation, industrial revolution has been accompanied by NDE revolution such that new methods of NDE have been introduced and new ways of applying these methods have been developed. Through the new technologies in NDE 4.0 high-performance and adequate measurement tools will be available worldwide while the communications and connections through the internet allows the involvement of specialists for discussion and decision making. Meanwhile NDE 4.0 is still opening its way into daily applications, the future is shaping by a combination of human and smart machines working together based on real-time evaluation, computation and communications which is known as NDE 5.0. The revolution of NDE over the time has similar steps as the industrial revolution such that one can correlate these steps and see the similarities such as summarized in Figure 10.

The concept of NDE 4.0 (and NDE 5.0) becomes more prominent when considering the advancement in material development and manufacturing processes. One of these recent advanced technologies which implies the crucial need for developing new measurement methods and NDE techniques is additive manufacturing [44]. Due to the intricacy of manufacturing process in AM techniques, as well as geometrical complexity of the produced parts and surface finish condition [46], new NDE and metrology methods have to be developed for quality and maintainability of AM parts. Modeling and simulation applications in NDE become even more essential in NDE 4.0 since they can provide crucial information on NDE experimental design, integration of NDE in design and manufacturing processes and interpretation of NDE results [47,48,49].

## 4. Digital Twins in NDE

**Figure 11:**
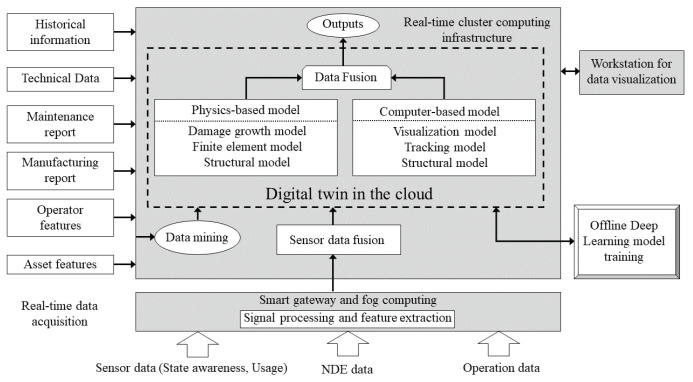
System architecture of digital twin ecosystem (After [50]).

The potentials and capabilities of Multiphysics modeling and data-driven analytics as described above are the basis for the “Digital Twin” concept [50]. Using digital twin, a live digital representative (model) of a system can be generated. The immediate advantage of such a model is that it can continuously adapt to the operational conditions using the in situ monitoring, collection and processing of the associated sensors’ data on the system. As a consequence, digital twins can predict the future of the corresponding physical counterpart and thus become a core part of the proactive and predictive maintenance plans. Since in situ condition monitoring of the systems usually contains multiple data sources (also due to advancement in sensors and data recording devices), data fusion has a key role in the digital twin framework. Data fusion integrates multiple data sources to produce more consistent, accurate, and useful information and enhances the flow of information from raw sensor data to high-level decision making steps. Signal-, feature-, and decision-level fusions are generally integrated into the digital twin framework. Figure 11 shows the system architecture of the digital twin ecosystem.

In the case of NDE, integrating the data from different steps in the digital twin of the inspected part can be used in future as a baseline for structural health monitoring and predictive maintenance plans of the part. In addition, it can be used to determine the important parameters of remaining useful life (RUL) of the physical twin. The other useful applications of digital twin in NDE are the capabilities of early warning, anomaly detection, prediction and optimization. This has direct economic impact due to implementation of predictive maintenance by turning the recorded data and information into actionable decisions.

## 5. Virtual Reality (VR) and Augmented Reality (AR)

By advancing NDE techniques and application of AI/ML algorithms for NDE interpretation and evaluation, the role of other relevant technologies become even more important. As an example in the core area of remote monitoring and evaluation, Virtual Reality (VR) and Augmented Reality (AR) tools can be implemented by developing adequate portable inspection devices and modern human machine interface (HMI). The large amount of data and metadata generated in NDE is the main challenge in management, assessment and visualization of the NDE results. Thus, the available information may not be fully utilized and cause less than optimal decision making or inaccurate decisions. Therefore there is a crucial need for a novel strategy such that data is not separated from their environment but becomes part of it. VR/AR can be a promising and suitable tool to address the above challenges. The interaction between the users and VR/AR framework provides the input for use of these technologies in NDE. As examples of practical work which has been done on application of VR/AR in NDE, Ababsa [51] showed how a more appropriate 3D visualization tool can be developed by AR to improve the understanding and interpretation of NDT data obtained from a nacelle of an Airbus A380. In Ababsa’s study, a set of data including the nacelle CAD file, coordinates of the ultrasonic transducers and their locations on the nacelle, and the simulation files containing defect coordinates have been used for the aims of visualizing the sensors used for the NDE measurement on the real structure, the defect region of the structure, and accurate localization of the defect. The experimental tests have shown that Ababsa’s developed application works reasonably well however the reported challenges were that the model had to be simplified to avoid overloading the computations of the augmented scene and also the tracking becomes unstable sometimes in closer locations to the structure. Nevertheless, the promising results showed that a more in-depth study can resolve these issues and provide a better VR/AR environment for NDE applications.

## 6. Challenges and Needs Assessment

### 6.1. General

General challenges in application of AI methods for NDE are that usually a sufficiently large set of training data, a high signal-to-noise ratio, and an optimized and exact fixation of components are required [11]. In addition, it should be noted that for accurate performance of AI techniques for NDE, an adequate level of theoretical and mathematical knowledge, experience in applying NDE techniques for inspection and material characterization, and carefulness is necessary [52].

### 6.2. Preprocessing

NDE examinations usually provide a large amount of data which requires prior knowledge about the NDE technique which has been used, material specifications, experimental setup, and equipment specifications (calibration procedure, sampling frequency, digitization capabilities, signal to noise ratio, etc.). Preprocessing of NDE data is usually required to prepare raw data for further analysis and specifically to use in AI algorithms. In data preprocessing, it is desired to smooth out insignificant features while allowing relevant important features to survive. These preprocessing procedures can be challenging and time-consuming tasks. As one of the reasons, since the AI algorithms are very sensitive to minor shifts in the features, they may map the same target into different features.

### 6.3. Physical Situation

Integrating the knowledge about the part under inspection with NDE and its physical situation is another need for the ultimate goal of automated defect detection using AI. This is because in NDE, the same signal or image can mean different things. As an example, an ultrasonic echo or a contrast change in radiography image can indicate a designed drilled hole in a part or it can be an indication of a defect in another situation. This indicates the need for a knowledge-intensive AI-based NDE model to integrate the physical condition of the materials into account for automated defect detection. This is where the concepts and applications of the modeling, simulations and digital twins for NDE become major role players.

### 6.4. Opportunities

AI and ML techniques provide a powerful approach to NDE and SHM applications in terms of useful algorithms and fast and accurate analyses of very substantial quantities of data that are acquired by recent and advanced NDE/SHM techniques.In addition to the benefits and advantages that AI/ML techniques provide for traditional NDE/SHM applications, they are demonstrated to be successful for in-line inspection and in situ process monitoring applications in many industries and manufacturing facilities.Last but not least, since the next generation of NDE will be based on data, automation and digitization, NDE 4.0 is considered to be the future of NDE as a central tool of quality assurance.

## 7. Summary and Conclusions

This paper describes and reviews the role and applications of the recent advancements in data science and technologies including AI/ML, AR/VR and digital twin in the crucial field of NDE. These technology advances and requirements have a great impact on how conventional NDE is being done and already started changing the traditional way of material testing and evaluation. In addition, NDE techniques are also advancing in recent years due to advancements in electronics, extensive research on physics of the problems, as well as the need for adopting NDE methods for new materials and manufacturing processes. Moreover, using simulation approach, a large training data set can be generated which enables adequate application of AI/ML techniques such as deep learning for crack characterization. As discussed above in this paper, several investigations showed that the percentage of accurate defect detection and characterization (such as sizing) can be doubled when using AI/ML techniques such as CNN compared to traditional techniques such as 6-dB drop [53]. All these technology advances will change the future NDE and maintenance, repair, and overhaul (MRO) business in almost every industry. Table 1 provides some examples of studies on each AI/ML technique in NDE applications.

## Figures and Tables

**Table 1:** Machine Learning Algorithms with NDE.

Algorithm	NDE Methodology	Paper Reference
K-Means Algorithm	Acoustic Emission Signal	[16,25]
	Thermal Imaging	[17]
	Pulse Eddy Currents	[18]
	Thermography	[54,55]
DBSCAN	Ultrasonic Testing	[20]
	Ultrasonic Lamb Wave	[46,55]
	Impact Echo, Ultrasonic Pulse Echo	[56]
	Laser Ultrasound	[57]
Spectral Clustering	Terahertz Spectroscopy	[23]
	Vibration Signals	[58]
	Spectral Kurtosis	[59]
Hierarchical Clustering	Ultrasonic Echo Testing	[24]
	Electromechanical Impedance Method	[59,60]
Association Analysis	Fiber Optic Sensors	[25]
	Multi-point Laser Vibrometers	
	Acoustic Emission Sensors	
Support Vector Machine	X-Ray Casting	[28]
	Long Range Ultrasonic Testing	[29]
	Raman Spectroscopy	[61]
K-Nearest Neighbor	Microwave Testing	[30]
Neural Networks	Ultrasonic Pulse Velocity Test	[34]
	Thermograms	[35]

## Data Availability

Not applicable.
